# Postnatal development of the subarcuate fossa and subarcuate canaliculus—a computed tomographic study

**DOI:** 10.1007/s00276-018-2045-x

**Published:** 2018-05-29

**Authors:** Mateusz Maślanka, Tymon Skadorwa, Bogdan Ciszek

**Affiliations:** 1 Department of Descriptive and Clinical Anatomy, The Medical University of Warsaw, 5 Chalubinskiego St., 02004 Warsaw, Poland; 2 Department of Pediatric Neurosurgery, Bogdanowicz Memorial Hospital for Children, 4/24 Nieklanska St., 03924 Warsaw, Poland

**Keywords:** Subarcuate fossa, Subarcuate canaliculus, Petromastoid canal

## Abstract

**Purpose:**

The subarcuate fossa (SF) is an anatomical structure situated on posterior wall of the petrous part of the temporal bone. In older children and adults, SF is a shallow depression and the subarcuate canaliculus starts within it. Awareness of postnatal changing morphology of this region is important especially for otosurgeon. The aim of this paper is to characterize both SF and SC by means of anatomical and radiological methods.

**Methods:**

The study was carried out on CT scans of 101 children, aged 1–60 months. Length of the pyramid (PL), the distance between the anterior semicircular canal (ASC) and the pyramidal apex (PLM), the outer diameter of ASC (ASCD), width under ASC (SFWM), the distance between the fundus of SF and ASC (SFLL), the maximal width of SF lateral to ASC (SFWL), the distance between the fundus of SF and posterior surface of the pyramid (SFL) were measured.

**Results:**

Average value of all measured distances: PL 52.14 ± 6.32 mm and PLM 25.73 ± 3.47 mm (raised with age); ASCD 8.63 ± 0.67 mm; SFWM 0.95 ± 1.24 mm; SFLL 1.07 ± 1.63 mm; SFWL 0.76 ± 1.19 mm; SFL 3.60 ± 2.50 mm.

**Conclusions:**

Petrous part of the temporal bone grows with age up to 5 years old, whereas ASC does not. SF diminishes with age: lateral to ASC is well developed in newborns and infants (up to first year), rapidly diminishes in children aged 1–2 years and is totally absent in children > 2 years. SF medial to ASC is constant and diminishes with age. In children older than 3 years morphology of SF is similar to adult.

## Introduction

The subarcuate fossa (SF) in adults is a shallow depression on the posterior surface of petrous part of the temporal bone. It is situated superior and lateral to the internal acoustic meatus. It has a shape of real fossa in most of the mammals. In tree climbing animals, such as small apes (prosimians, macaques), it is of relatively large volume and houses the petrosal lobule of the cerebellar paraflocculus. On the other hand, in greater apes, living mostly on the ground (gorilla, chimpanzee, orangutan) and in human, the volume of adult form of SF has been highly reduced [4].

The bottom of SF usually gives an origin to the subarcuate canaliculus (SC), also known as petromastoid canal or antrocerebellar canal of Chatellier [9]. It connects the posterior cranial fossa with periantral mastoid cells. Both SF and SC house the dura mater and subarcuate blood vessels that supply surrounding tissues and mastoid cells [5]. One of these vessels, the subarcuate artery, usually originates from the anterior inferior cerebellar artery or from the labyrinthine artery, whereas the subarcuate vein drains into the superior petrosal sinus or directly into the sigmoid sinus [11, 13]. The role of SF and SC has not been clearly explained in the literature. Some reports provide the hypothesis that the SC can be a potential route for infection from the middle ear to the posterior cranial fossa—even 3.2% of cerebellar abscesses can have such origin [9].

The reason of above may be explained by developmental characteristics of SF. Throughout the intrauterine period, SF develops together with the membranous labyrinth under the arch of the anterior semicircular canal (ASC). It may be distinguished in 11 Hbd human fetuses—as a hollow pouch of 2 mm diameter, which normally begins to ossify after 15th week of gestation [10] and attains the greatest dimensions between the 24 and 28 weeks. After this stage, the SF slowly but constantly decreases in size, although it is still relatively big after birth [4]. Some authors hypothesize that the size of FS in early stages of postnatal development may promote spreading infections into the cranial cavity. On the other hand, the dimensions of SF may also serve as important landmarks for the purposes of planning the implantation of cochlear implants in the youngest children [8].

Subarcuate fossa was described several times by other authors and the differences in SF anatomy between the population younger and older than 5 years have been described [5–8]. However, anatomical descriptions of SF in individuals younger than 5 years given in the literature are usually incomplete, due to a fact that this period is characterized by the greatest intensiveness of the development of the petrous part of the temporal bone. Therefore, the aim of this paper is to characterize both SF and SC by means of anatomical and radiological methods.

## Materials and methods

The study was carried out on anonymized CT scans (bone window) of children of both sexes collected retrospectively at the Bogdanowicz Memorial Hospital for Children, Warsaw, between February 2011 and January 2012. All the scans were made due to clinical indications with CT scanner Siemens Somatom Emotion (slice thickness from 0.75 to 3 mm, the exposition performed with source voltage of 270 kV and current of 100 mA). Only the scans of patients aged 1 month–5 years (60 months) were included in the study. Patients with congenital or acquired malformations of the head or the history of such in their medical records, the scans containing fractures of the middle or posterior cranial fossa or inflammatory disease of the temporal bone were not included in the study. Finally, the studied group consisted of 101 cases (43 female, 59 male).

For a better clarity of results, the studied sample was divided into three age groups: A (from 0 to 12 months, 32 cases), B (from 13 to 30 months, 37 cases) and C (from 31 to 60 months, 32 cases).

**Fig. 1:**
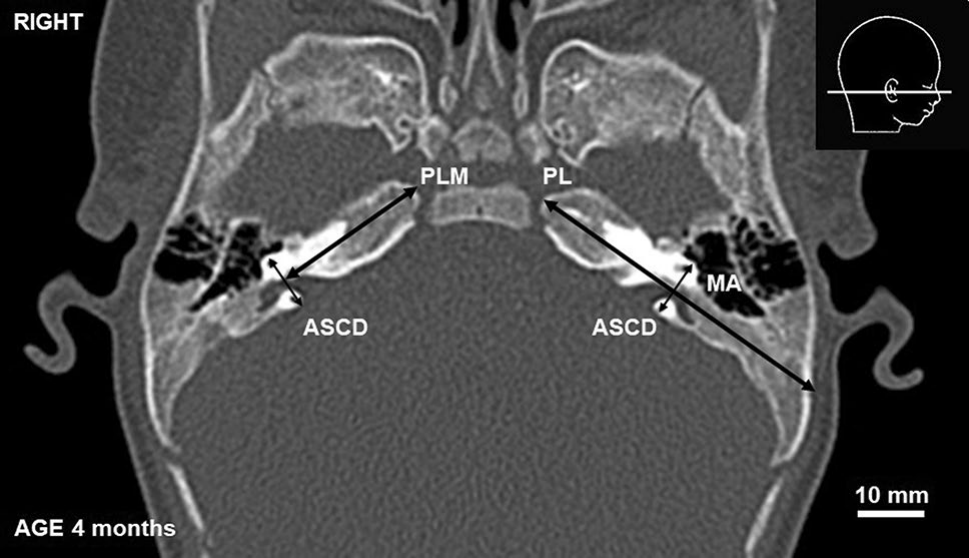
CT scan of a 4-month-old infant. *MA* mastoid antrum, PL, PLM, ASCD—parameters described in the text and Table 1

**Fig. 2:**
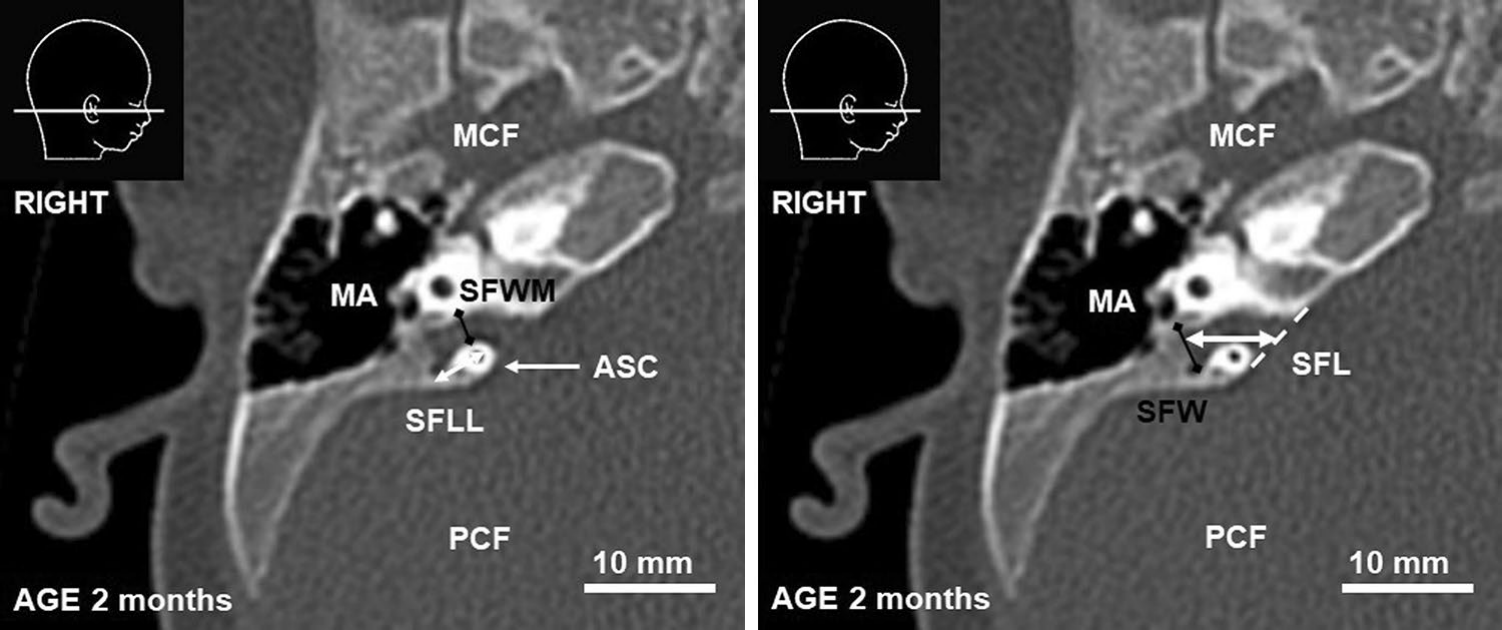
CT scan of a 2-month-old infant. *ASC* anterior semicircular canal, *MCF* middle cranial fossa, *MA* mastoid antrum, *PCF* posterior cranial fossa. SFLL, SFWM, SFW, SFL—parameters described in the text and Table 1

To describe relations between SF and petrous part of the temporal bone, several parameters were measured: (1) the length of the pyramid (PL), (2) the distance between the anterior semicircular canal (ASC) and the pyramidal apex (PLM) and (3) the outer diameter of ASC (ASCD) (see Table 1; Fig. 1). The changing morphology of SF was described by: (4) its width under ASC (SFWM), (5) the distance between the fundus of SF and ASC (SFLL), (6) the maximal width of SF lateral to ASC (SFWL), and (7) the distance between the fundus of SF and posterior surface of the pyramid (SFL) (see Table 1; Fig. 2).

**Table 1:** Description of parameters used in the study

Parameter (all measured in horizontal plane)	Description
PL	Length of the petrous part of TB measured from the apex to the external table of TB. The measurement was perpendicular to ASCD
PLM	Length of the petrous part of TB medial to the ASC measured from the apex to the ASCD. The measurement was perpendicular to ASCD
ASCD	Outer diameter of ASC measured between the most distal points of the lumen of ASC
SFWM	Width of SF under ASC (width of medial part of SF)
SFWL	Maximal width of lateral part of SF measured laterally to ASC
SFLL	Distance between the fundus of SF and ASC (length of the lateral part of SF). The measurement was perpendicular to ASCD
SFL	Distance between the fundus of SF and posterior surface of the pyramid (length of the whole SF). Measured in horizontal axis

*TB* temporal bone, *ASC* anterior semicircular canal, *SF* subarcuate fossa

**Fig. 3:**
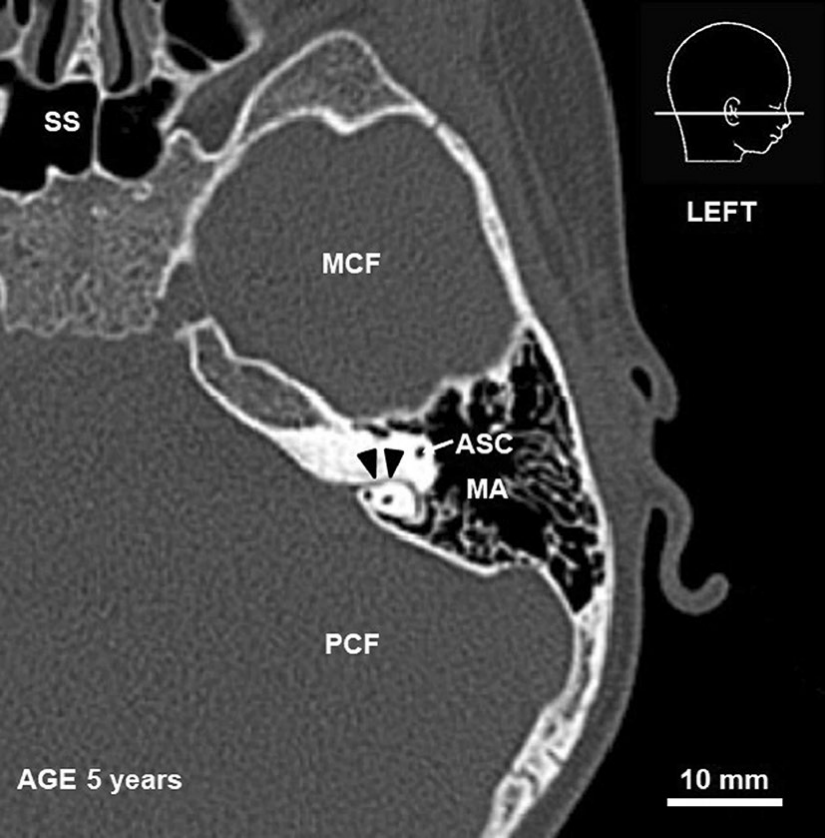
CT scan of a 5-year-old child. Arrowheads indicate subarcuate canaliculus, *ASC* anterior semicircular canal, *MCF* middle cranial fossa, *MA* mastoid antrum, *PCF* posterior cranial fossa, *SS* sphenoid sinus

The presence of the SC was noted in every case. It was identified as a bony canal of a diameter less than 2 mm, emerging from the fundus of SF below ASC and connecting the posterior cranial fossa with mastoid air cells (Fig. 3).

All the measurements were obtained with the use of software provided and calibrated by the manufacturer. The parameter values were noted in a database designed for the purpose of this study. Obtained data were analyzed statistically with the use of StatSoft Statistica 13.1 software. We used parametric tests (*t* test and Pearson correlation coefficient) due to normal distribution of our data.

## Results

**Fig. 4:**
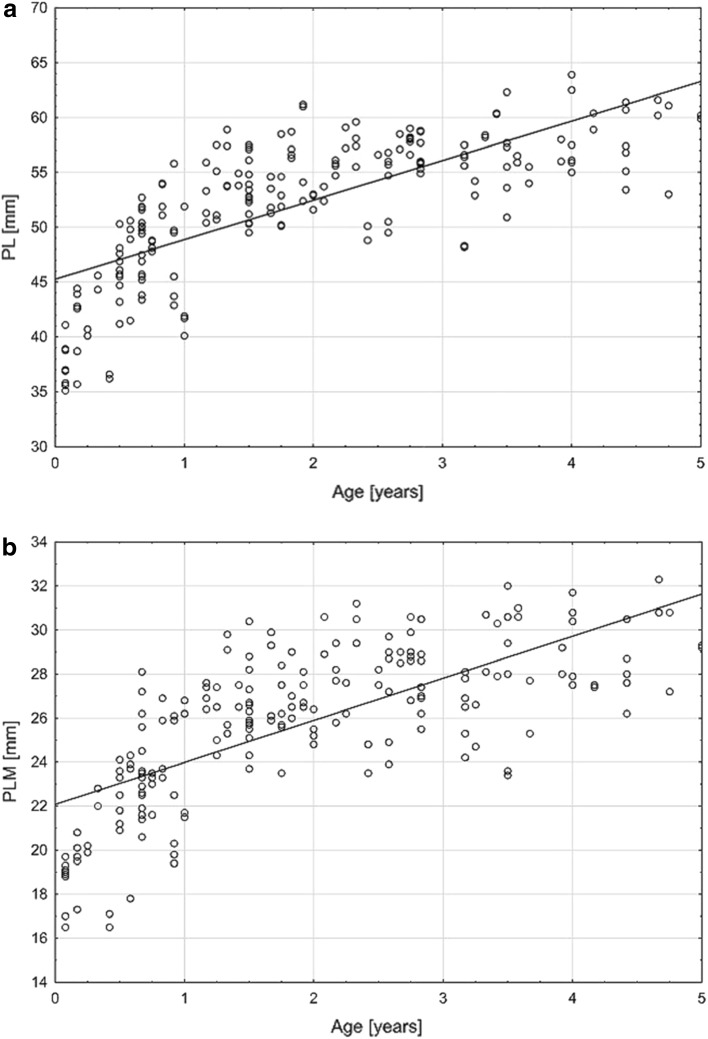
Chart of correlation between the length of petrous part of the temporal bone and age. PL, PLM—parameters described in the text and Table 1

Obtained data showed that both PL and PLM constantly increase with age (Fig. 4a). Within the studied period of 60 months, the length of the pyramid ranged from 35.1 to 63.9 mm and the PLM distance increased from 16.5 to 32.3 mm (Fig. 4b). Both these parameters revealed a strong positive correlation with age (PL Pearson’s *r* = 0.74, *p* < 0.05; PLM Pearson’s *r* = 0.73, *p* < 0.05). On the other hand, the ASCD showed no correlation with age; its value was more constant throughout age groups and ranged from 6.7 to 10.5 mm (Table 2).

**Table 2:** Descriptive statistics of direct parameters used in the study

Parameter (mm)	Group A (0–12 months)				Group B (13–30 months)				Group C (31–60 months)			
	Avg	SD	Min	Max	Avg	SD	Min	Max	Avg	SD	Min	Max
PL	45.44	5.27	35.10	55.80	53.81	3.90	40.10	61.20	56.83	3.20	48.20	63.90
PLM	21.94	2.72	16.50	28.10	26.82	1.96	21.50	31.20	28.20	2.08	23.40	32.30
ASCD	8.60	0.72	6.80	10.50	8.64	0.66	6.70	9.60	8.65	0.63	7.10	10.20
SFWM	2.43	0.88	0.70	4.20	0.51	0.82	0.00	2.40	0.00	0.00	0.00	0.00
SFWL	3.13	1.25	0.00	5.70	0.23	0.72	0.00	3.60	0.00	0.00	0.00	0.00
SFLL	6.61	1.27	2.90	9.60	3.03	1.60	0.90	7.60	1.32	0.64	0.80	4.50
SFL	2.16	1.12	0.00	5.30	0.22	0.57	0.00	2.30	0.00	0.00	0.00	0.00

*Avg* average value

**Fig. 5:**
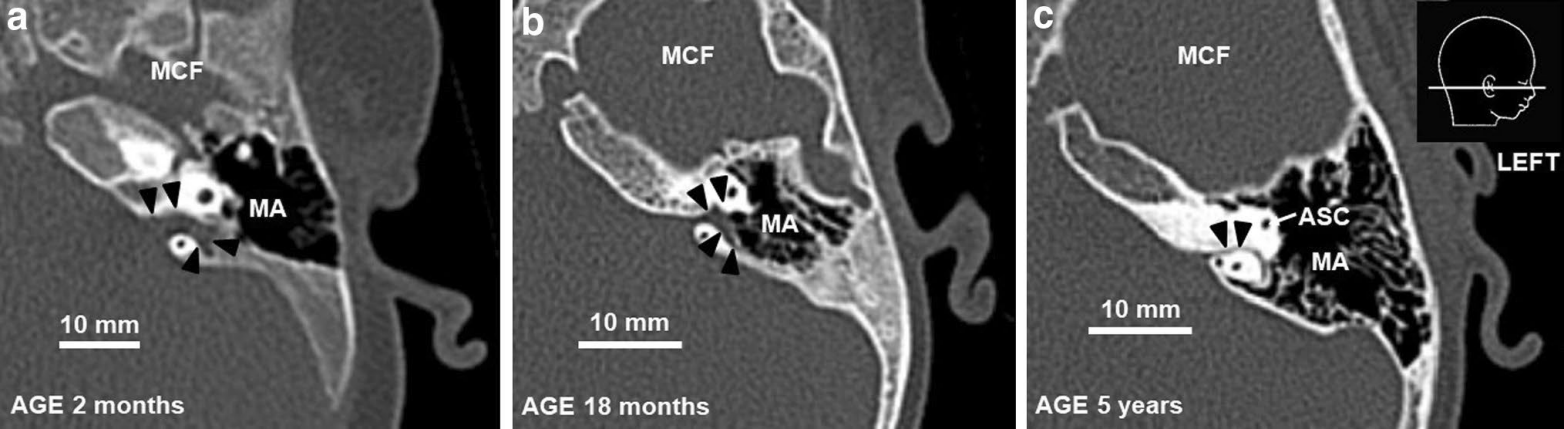
Morphological changes of SF with age. **a** SF of 2-month-old infant (group A), **b** SF of 18-month-old child (group B), **c** morphological transformation of SF into SC (group C). Arrowheads indicate SF and SC, *ASC* anterior semicircular canal, *MCF* middle cranial fossa, *MA* mastoid antrum

**Fig. 6:**
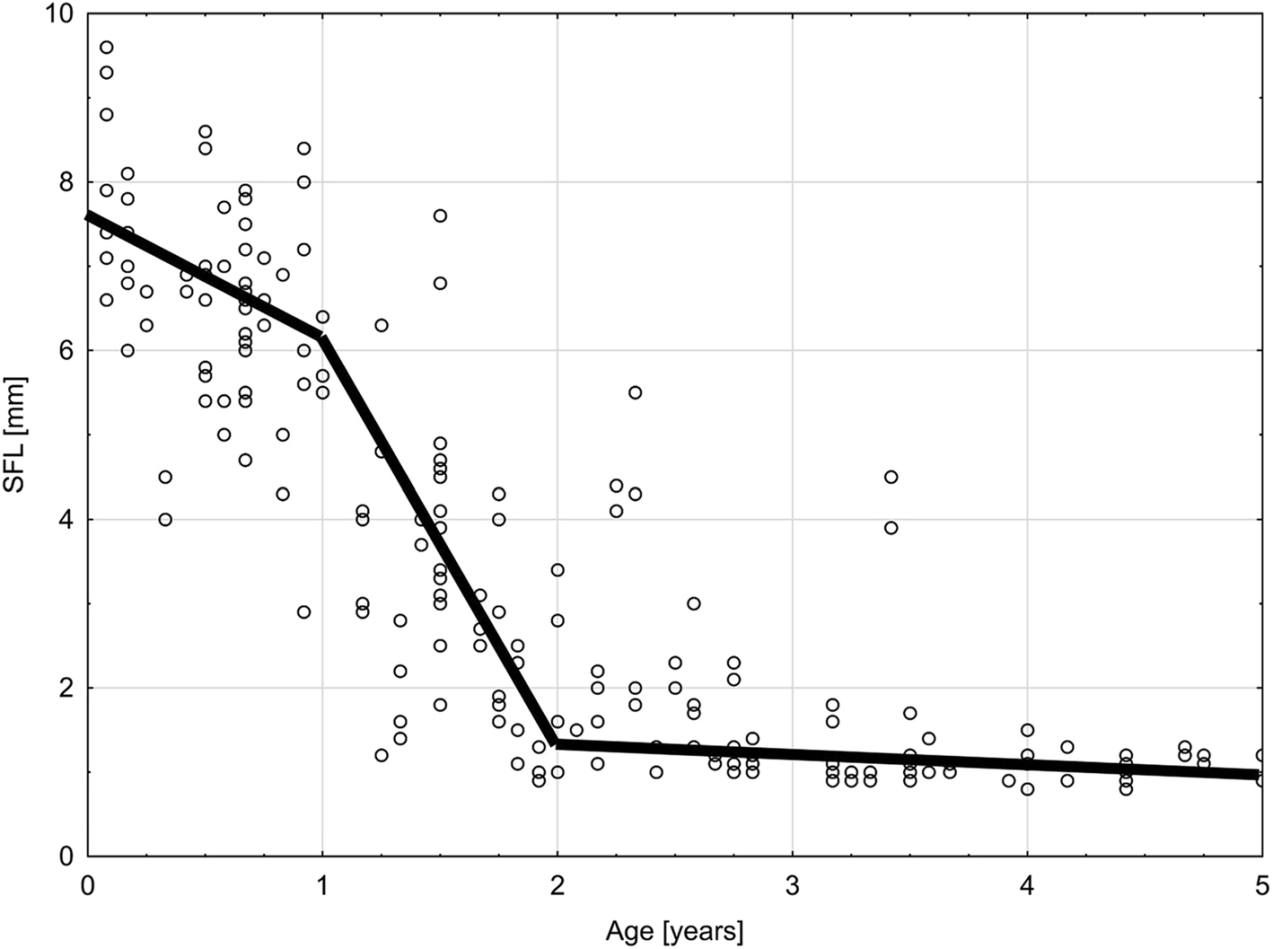
Chart of correlation between the length of SF (SFL) and age. Group A (from 0 to 12 months), group B (from 13 to 30 months) and group C (from 31 to 60 months)

The morphology of SF revealed a strong relation to the measured parameters: its portion located lateral to the ASC decreased rapidly in all measured dimensions and was totally absent in children older than 18 months (Fig. 5). The dynamics of the reduction of SF volume can be precisely followed based on the changes in SFL (Fig. 6). In group A, the average depth of SF was the greatest, in group B it decreased to almost total absence in group C. As shown in Fig. 6, this parameter attains a constant average adult depth of 1 mm after 36 months of life.

The subarcuate canaliculus was identified in 13 out of 64 temporal bones in group A (20.3%), in 58 out of 74 temporal bones in group B (78.4%) and in 55 out of 64 temporal bones in group C (85.9%). All the measured parameters are presented in Table 2.

## Discussion

The petrous part of the temporal bone is characterized by a complex growth pattern. The descriptions of such can be found in numerous papers [2, 5, 10]. The computed tomography seems to be an accurate method for assessment of the temporal bone development, as it shows age-specific stages of internal bone organization and, therefore, allows a precise estimation of appropriate timeline for each stage [5–8, 10, 13].

A decrease of SF after birth remains a fact and the differences between children up to 5 years and older have been well established [6–9, 13]. Proctor indicates that SF narrows into the canal before the 5th year of life and the adult morphology of SF is attained in the 5th year of life. Migirov and Kronenberg support the findings of Proctor and do not find significant differences in SF morphology between older children and adults. However, the postnatal developmental pattern of SF in children below the age of 5 years is more complex and, in our opinion, should be described more precisely.

After birth, the petrous part of the temporal bone grows rapidly during the first 2 years of life, especially in long axis, and this pattern of growth promotes ASC (together with SF) to take the central position in the pyramid. It seems interesting that the ASC can serve as a marker of the symmetry of the temporal bone development—based on its position the growth of the petrous part splits into two alternative pathways. The portion of the pyramid medial to ASC grows as a solid bone, by increasing in all the dimensions, whereas the portion lateral to ASC gains its size by the enlargement of pneumatic areas. Meanwhile, the bony labyrinth seems to be unaffected by the growth of surrounding structures [2, 5].

In our study, in children younger than 12 months the volume of SF is the greatest in postanatal life. The appearance of SF in group A refers to type IV of SC by Migirov and Kronenberg [7, 8]. Our distinction of SF into two portions located on both sides of the ASC allows to describe the decreasing of SF in a more precise manner. In our sample, the portion lateral to ASC is well developed in newborns and infants, reaching its maximal depth in the first month of life. It decreases throughout the first half of the second year to finally disappear about the 18th month. This observation correlates with the paper of Hilding [5]. Contrarily, the portion of SF medial to ASC seems to decrease less rapidly, as it is still present throughout the second year of life and attains the adult depth (about 1 mm) in the third year of postnatal life (group C, Fig. 6), which is earlier than about 5 years, stated in other papers [8, 9].

**Fig. 7:**
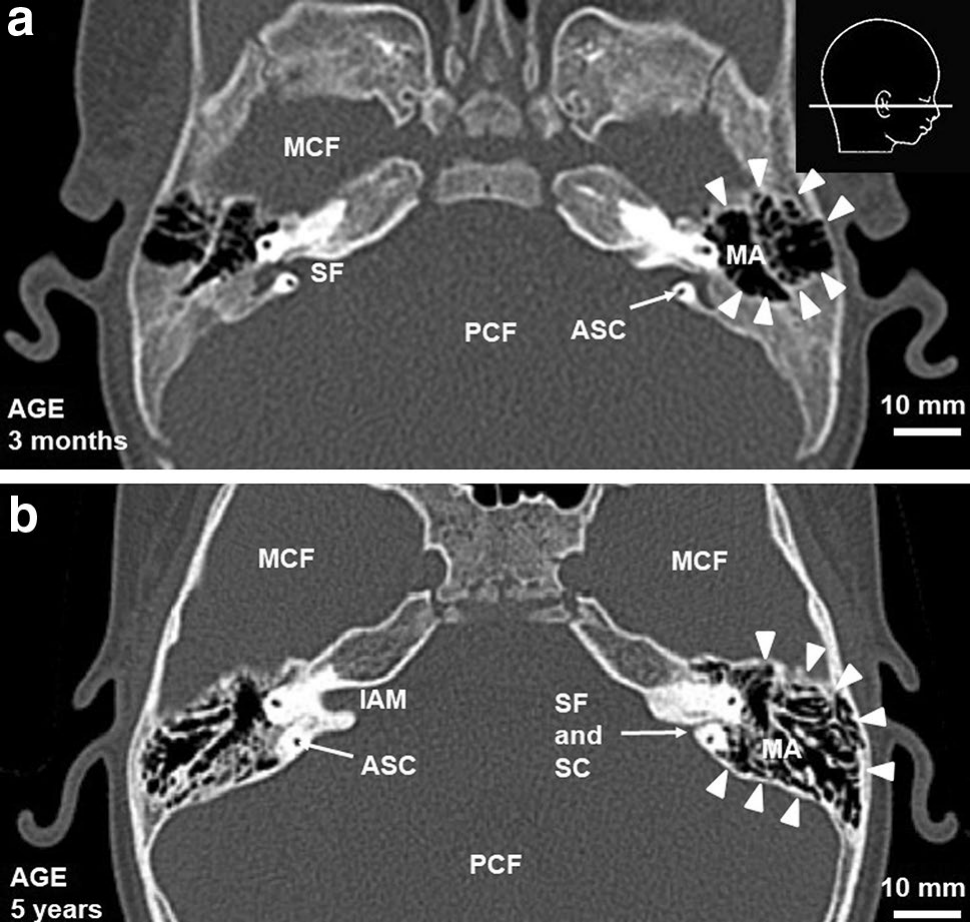
Morphological changes of pneumatization of petrous part of the temporal bone with age. **a** Temporal bone of 3-month-old infant, **b** temporal bone of 5-year-old child. Arrowheads indicate pneumatization of temporal bone, ASC anterior semicircular canal, MCF middle cranial fossa, PCF posterior cranial fossa, MA mastoid antrum, IAM internal acoustic meatus

The postnatal development of SF may result in problems with its proper description, especially in terms of distinction between the SF and SC. This may be one of the limitations of this study. It seems that SF decreases and narrows into SC. This transformation starts laterally and is related to the pneumatization of the mastoid process (Figs. 5, 7) [7, 8, 14]. It is important to note that a standard calibration of CT scanners used for clinical purposes does not allow exact measurements of the subarcuate canaliculus, as it is limited to ossified portions of the structures described in this study. Therefore, we decided to apply a criterion of the width of bony canal to differentiate the SF from SC. The structure wider than 2 mm was identified as the SF, whereas the canal narrower than 2 mm was considered the SC. This is coherent with other reports as well as requires the use of slices of appropriate thickness. As shown in other papers, a 1-mm slice is appropriate to visualize SC in all cases, whereas the thickness above 2 mm may be insufficient for this purpose [6, 12]. As in other papers, all the recognized SC had a form of a single canal [7, 8, 13]. Regarding this problem, it seems that a targeted study using more accurate imaging techniques would be most appropriate to evaluate this structure. Since experimental configuration of CT scanner raises ethical concerns, in our opinion further study might be possible with the comparable population of pediatric cadaveric specimens and the use of CT scanner of a greater accuracy (i.e. cone-beam CT).

Developmental morphology of SF and SC contributes to broadening of clinical experience. The anatomy of these two subarcuate structures and their radiological appearance are essential in recognizing the lines of temporal bone fracture in case of padiatric head trauma [1, 6]. The knowledge about them is crucial in diagnostics of cerebrospinal fluid fistulas, that may originate from SF [3, 13], especially as a potential complication of cerebellopontine angle surgery. Other complications, such as unintended damage to the subarcuate artery, might be prevented with a detailed CT-based preoperative planning, which would also benefit from this study.

Finally, the morphology of SF and SC seems to prove that infections can spread from the tympanic cavity or mastoid air cells, especially in the first year of life. Hilding in his paper indicates that the infection on its way can affect only soft tissue without destroying the bone [5]. The results of our study seem to concur with this opinion. The width of the canal and its contents may promote the transmission of pathogens with the easiest route being probably through the sinusoidal, low-pressure-type, petromastoid vein [11].

## Conclusions

The SF in newborns and infants is a relatively wide depression that narrows into the subarcuate canaliculus, connecting the mastoid air cells with the posterior cranial fossa. After the 12th month of postnatal life, its portion lateral to ASC starts to decrease, by gradually losing its previous dimensions to the complete absence in the 18th month. The portion of SF located medial to ASC remains present but is reduced to adult size in the third year of life.
